# American Dental Association

**Published:** 1887-09

**Authors:** 


### AMERICAN DENTAL ASSOCIATION.

### TWENTY-SEVENTH ANNUAL MEETING.

REPORTED FOR THE INDEPENDENT PRACTITIONER.

The twenty-seventh annual meeting of the American Dental Association convened at the Park Theater, Niagara Falls, August 2, 1887. The meeting was called to order by the President, Dr. W. W. Allport, of Chicago, at 11 o'clock A. M.

The reading of the minutes being called for, Dr. Shepard moved that, as they had already appeared in print, the reading be dispensed with, and the motion was declared carried. The secretary, however, as a report of the board of officers, read the correspondence concerning the change of the place of meeting, the protest of Drs. Barrett and Abbott against the abandonment of the meeting, and the final decision of the board, that the meeting should be held at Niagara.

The roll was called by the treasurer, and a large number of the permanent members were found to be present.

The reports of the various committees and of the officers were presented, and the annual grist of amendments to the constitution was ground out. A number of these were presented at the meeting of 1886, chiefly relating to the duties of the Executive Committee, enlarging the powers of the chairman, etc., and they had necessarily been laid upon the table for one year. They were all rejected save one, which gave the chairman authority to call a meeting of the board at the request of two of its members. The number was changed to five, and it was then adopted.

The Treasurer presented his annual report, which showed a balance on hand of $2829.64.

Both Vice-Presidents being absent, the President called Dr. Crouse to the chair and read the President's annual address. It was largely historical in character, was of considerable length, and hence no abstract could do it justice. There were a number of excellent suggestions for the society in it, and it showed a great deal of care and research in its preparation. It was heartily cheered upon its completion.

Dr. Atkinson took exception to some of its statements of fact, and objected to giving it to the world as an expression by the Association. He offered a resolution that it be referred to a Special Committee for revision, but a substitute that it be referred to the Publication Committee, as usual, was carried almost unanimously.

TUESDAY EVENING SESSION.

After the usual preliminary business, the report of the Committee on Credentials, etc., Section II—Dental Education, Literature and Nomenclature—was called, and the report was presented by the secretary of the Section, Dr. Ottofy. The report was an exhaustive one, and its reading occupied more than an hour, but it was so thorough and completely systematized that it was listened to with unflagging interest. The condition of educational affairs in the different countries of the globe was recapitulated, and the legal and professional status of dentistry considered. In adverting to educational matters in this country, the report spoke of the good work that is being done by Meharry College, and recommended that additional facilities be furnished colored students of dentistry, that they may be enabled to minister to the wants of their own race. The new books and journals of the year were reviewed, and the status of our literature set forth.

Two papers were presented from the Section, one on Nomenclature, by Dr. Atkinson, and the other on Early Education, by Dr. Crouse.

The first paper was read by its author, and was a plea for a revision and proper classification of professional terms.

The second paper was likewise read by its author, and was largely given up to a consideration of the advantages to be derived from technological study.

The essayist said that education is understood to be the accumulation of knowledge. But too often the young man who has secured his diploma from a college, the possession of which is supposed to fully qualify him for his life's work, is practically without education. He may have a reputation for scholarship, but the knowledge that is most essential for practical success in life has not been acquired. He may have literary training, but his instruction in the practical arts has been entirely neglected.

Kindergarten schools originated in the endeavor to train children in manual labor. It was found that such occupations tended to strengthen and develop the mind. It was not alone the hand that was employed, but the head was also engaged. The development of the kindergarten theory in education bids fair to revolutionize the educational affairs of the world. Our dental colleges are but an elaboration of this idea, for they train the mind and hand together, and this will account for the rapid development of dental educational matters.

DISCUSSION.

*Dr. Atkinson*—When, in the presence of so many teachers as are here present, there is silence upon such a subject as this, it teaches us its vastness. The review of Dr. Ottofy is a labored one, and for it I return my most hearty thanks. If we could once grasp the idea that is next us, we should have made great advance. The sober second thought is of Satan, always. We have heard something of ptomaines. They have been known to exist for a long time, but the experts who were searching for the causes of certain changes were all the time looking for some mineral salt that was inimical to the body. They never conceived the possibility of the formation of an alkaloid that should induce a retrogressive metamorphosis. The presence of this alkaloid will account for many changes which have been attributed to living organisms.

*Dr. Abbott*—In his excellent paper, Dr. Ottofy maintains the superiority of German schools over all others, and attributes this to the asserted fact that Government appoints the teachers. To this I take exceptions. In Berlin there lives one man who has charge of all the schools, and naturally he likes to surround himself with men who are in accord with him, and who will advance his own ideas and theories. In a German University no student is permitted to receive instruction from any one save the regular professor. In the dental department of the University of Berlin, but one set of ideas can be inculcated. No clinics in operative dentistry are permitted, save those which illustrate the special teachings of the regular professor of operative dentistry. This tends to a narrowness and exclusiveness in practice. The American people have more knowledge and a better appreciation of dentistry than any other in the world, and the result is that our dentists are encouraged to yet higher attainments. We are making continual advancement, and by means of our clinics and societies the knowledge is generally disseminated. In our schools, operators of widely differing methods are invited to give clinics, and thus the students have the advantage of a comparison of all methods, and are enabled to select the best, or that which is best adapted to their capabilities.

In other countries the people must be educated to an appreciation of high class dentistry before there will be the same skill exhibited by operators that there is here. You may travel Germany over, and you will scarce find a practicing native dentist who is competent for the class of operations performed by our average country dentist. And yet they affect to look down upon American dentistry. Often without sufficient knowledge of their business to be competent judges of what constitutes a good operator, they assume all the airs of virtuosos. Unable to insert even a fourth-rate amalgam filling, they talk learnedly, and assume to sit in judgment upon men whose operations they cannot even comprehend. If among them—and Germany is not alone in this thing—a progressive man gets a new idea in his head, no opportunity will be allowed him to exhibit it in a practical manner, but he will be summarily squelched for daring to be progressive.

*Dr. Winder*—Concerning nomenclature, it is difficult for language exactly to express just what is the conception of an idea. Pathologists endeavor to use a language peculiar to themselves, and they are continually introducing new terms. It is impossible to reduce to a system a language that is thus continually undergoing changes.

There has been a disposition on the part of some to make our system of education conform to European standards—to so alter our whole method of teaching that it shall comply with the requirements of the laws of foreign nations. That is exactly what we do *not* want. American dentistry certainly is not inferior to that of any other people, and we cannot make American dentists without giving prominence to the practical part of dental education, any more than playing upon the violin can be imparted by didactic lectures. The secret of the action of foreigners towards American dentists is that they are afraid of them, and cannot stand their competition, hence their only course is to fence them out.

*Dr. Guilford*—The report of the Section is very exhaustive. In the two papers presented there is a wide difference. One began at the very bottom, while the other was away out of comprehension, even at its commencement. The paper of Dr. Atkinson may be understood by him, possibly, but I doubt if any one else has any comprehension of what it was all about. The paper of Dr. Crouse is too elementary. It might possibly have place before a local society of neophytes, but it is scarcely adapted to a body that pretends to be scientific, like this. The education of children by handicraft is all very well, but we should remember that all this is elementary, and that really educated men are called upon to consider abstract things. When one has attained what is called a liberal education, his mind, his comprehension is broadened, and he is enabled to grasp any subject with greater facility. The trained mind makes a better hand, and the broadly educated man is always the better mechanic, other things being equal. An ignorant man is, under no circumstances, the better for his lack of discipline, while a liberal, even a classical education, makes a man the better qualified for any position in life.

*Dr. Crouse*—I should be more surprised at the lack of comprehension of kindergarten training, did I not know how wide-spread is the ignorance concerning it. Few, even among teachers, know what it is. It is a system of actual demonstration, rather than of memorizing. The usual college course is a mere matter of fashion. I have asked many college graduates if they had made any use of their college training, and they invariably answered, No. In the Polytechnic School of Boston, seventy-five per cent. of the graduates secure engagements before they close their training course.

*Dr. Taft*—I think the difficulty in our nomenclature arises from a lack of definite knowledge of the thing to be named, together with an ignorance of language. We find no trouble in exactly nominating anything with which we are well acquainted. The people whom we know intimately we can easily name, but those whom we have but casually met are not readily nominated, while those with whom we have no acquaintance we cannot name at all. Many of our misnomers arise from bad habits. We speak of the fang of a tooth, because we have associated with those who have miscalled it.

There are various phases of the educational problem. Dr. Crouse began, perhaps, on a lower plane than was necessary. We are more interested in technical than in preliminary education, and yet dentistry is at fault in permitting any one to enter upon a course of professional study before he has mastered preliminary science.

It is within the power of dentists to discourage those who would commence technical study without either natural or acquired ability.

We speak of training "students." Every progressive man is a student through all his life. The time of graduation is but the commencement of another course of study. No man's education is ever completed. There are in dentistry many men who were without early advantages for study. To those, a post-graduate course would be of the greatest advantage. These might be established in our colleges, and men who had been long in practice could go back for study, even if it were but for a month.

What shall I say about teachers? How many men make dental teaching the work of their life? At the best, it is usually but incidental. If our schools were sufficiently endowed to enable the teachers to devote their whole time to that pursuit, how much better might it not be for both schools and scholars?

WEDNESDAY MORNING SESSION.

After the transaction of the usual routine business, the consideration of the report of Section II was continued.

*Dr. Butler*—The report as read by the Secretary of the Section, while recommending that the college terms be lengthened to nine months, states that various excuses are made by the schools for the non-observance of the recommendation. If the colleges publish announcements promising certain things, the students are entitled to the benefit of all that is announced. Every matriculant is required to sign a kind of contract, promising faithful attendance, etc., but are not the schools themselves bound to give all that is pledged in the announcements? It is urged that there is not time for the special features promised. If this is the case, they should not be announced. Much good work is being done, but better is possible, and should be secured.

*Dr. Pierce*—The gentleman evidently has never served as the dean of a college. The burthen of the letters from students is, how short a time will suffice to put me through? Not how much can I learn, but how quickly can I obtain a diploma? We have a term of nine months, five of which are obligatory, and the other four are but poorly attended.

The purpose of the paper on primary education has not been properly understood. It marks an advance when the young are trained to habits of precision. I have had some experience in a school which received children that had been expelled from other schools, and I have observed that the most refractory were reformed by presenting a system in which they become interested. In this the punishments were deprivation of attendance at certain lessons, and it was effectual.

*Dr. Darby*—It would seem that educational advantages are not equally appreciated. Some students want all the avenues of information opened to them, while others only ask for just sufficient to obtain a diploma. In the seven months' course at the University of Pennsylvania, we lose students, because they can graduate at other institutions on a five months' course. We wish very much to prolong the course to nine months, and we only wait for the time when the students will accept it. It is the man who possesses a special fitness for any work who succeeds. Broad culture is desirable, but is not always attainable. We find men with excellent general training who have no adaptation. Others, with little schooling, but who have a special fitness, far excel them in life's work.

That which I desire especially to emphasize, is the readiness of the schools to advance the status as fast as the profession and the students will accept it.

*Dr. Atkinson*—Those of us who have been members of this society from the beginning, can mark the advance that has been made. I see before me many men who began very low. What has raised them to their present intelligence? Not the iron rules of the schools; not a thoroughly digested system, but practical work done outside the recognized text-books and school curriculums. The colleges assume that they have all the truth within an awfully restricted syllabus. It is individual and selfish ambition that fills the chairs in our colleges, and not the demand of the schools for men who are, by training and observation, best fitted for the positions.

*Dr. Barrett*—Permit me to say a few words concerning nomenclature. We are all aware of the embarrassment which at times overtakes us in the endeavor exactly to express what the mind has conceived. But in the majority of cases this arises, not from the barrenness of language, not from the paucity of terms, but from the multiplication of definitions, and the redundancy of names for the same thing. In the examination of an object, each of us sees some special phase or characteristic, and to that aspect as presented we desire to give a definite name. This special feature may be very clear to us, without having any meaning to the rest of the world, because they do not see it as we do. As an instance, Dr. Atkinson frequently refers to the revelations of "Angels" as the source of his particular ideas. This term has no significance to the rest of us. We look upon our conceptions as having their origin within us—as having been elaborated from our inner consciousness—and we have no use for "Angels" in this connection, or for other terms of Dr. Atkinson which express phases that he may possibly see clearly enough, but which have no existence for the rest of us. So in the attempts to express personal conceptions of things rather than the universally acknowledged standards, there is a multiplication of words, our nomenclature is loaded down with verbiage and confusion ensues.

Another source of perplexity arises from the fact that many of us are ambitious of using extremely technical language. We are fond of fire-new words, and of high-sounding terms. We are anxious to employ the latest invented phrase, and some of us seem ambitious to coin jaw-breakers, and thus get the start of every one else. I am sorry to see that at the present time there are no more "mechanical dentists." All are practicing "prosthetic" dentistry, and the good old term is relegated to the past. Every year at these meetings our vocabulary is enlarged, and new technical terms are added. One has been sprung upon us in this debate, and henceforth "metabolism" must take its place among us. What is the matter with the good old word "digestion?" In what respect is the new term better? It is this inventing of three or four words for one idea that brings confusion. The reform in our nomenclature should be toward simplicity, and not the endeavor to introduce yet more nominations where we already have sufficient.

Upon motion the subject was passed.

